# The Role of Neutrophil-to-Lymphocyte Ratio in the Diagnosis of Acute Appendicitis

**DOI:** 10.7759/cureus.51164

**Published:** 2023-12-27

**Authors:** Sundus A Khan, Raza Ashraf, Narmeen Hassaan, Mubashar Naseer, Muhammad Hassan Azad, Hamza Javed

**Affiliations:** 1 General Surgery, Northwest School of Medicine, Peshawar, PAK; 2 General Surgery, Shifa International Hospital, Islamabad, PAK; 3 Radiology, Ayub Teaching Hospital, Abbottabad, PAK

**Keywords:** hawthorn bias, receiver operating characteristic (roc) analysis, validity parameters, alvarado score, acute appendicitis, neutrophils to lymphocytes ratio

## Abstract

Background: Acute appendicitis (AA), a common reason for episodes of acute abdomen, is a surgical emergency. Its immediate diagnosis and management are of immense significance, as its diagnosis can become challenging at times, especially in resource-limited setups. The goal of this study was to ascertain the threshold value for the neutrophil-to-lymphocyte ratio (NLR) in diagnosing AA and to calculate the validity parameters for the NLR.

Methodology: A cross-sectional study was carried out involving 108 patients who were admitted to the surgical wards of Ayub Teaching Hospital, Abbottabad with suspicion of AA and subsequently underwent open appendectomy. Data was collected regarding the demography of the patients, physical examination findings, clinical presentations, and investigations including the histopathology and complete blood count, from which the NLR value was computed, and the Statistical Package for Social Sciences (SPSS), version 25.0 (IBM Corp., Armonk, NY) was utilized for the computation. Receiver operating characteristic (ROC) analysis was done to calculate the cut-off value of the NLR for diagnosing AA, and validity parameters were computed, taking into account statistical significance with a p-value < 0.05.

Results: Based on the ROC analysis, a threshold value for NLR indicating a positive appendectomy was determined to be 2.49 (sensitivity = 71.4% and 1-specificity = 12.5%) with an area under the curve of 90.6% (95% confidence interval {CI} 0.818-0.994, p<0.001). The sensitivity, specificity, and diagnostic accuracy of NLR for diagnosing AA were 71.43%, 87.5%, and 72.73%, respectively.

Conclusion: There is a strong correlation between NLR at a cut-off value of 2.49 and the diagnosis of AA. We suggest that NLR should be utilized as a complementary biomarker to clinical examination, aiding in the diagnosis of AA.

## Introduction

Acute appendicitis (AA), classified as a surgical emergency, stands out as the leading cause of acute abdominal pain [1]. The diagnosis of AA presents a serious challenge to surgeons due to the overlapping signs and symptoms with other causes of acute abdomen [1]. Delay in the diagnosis and surgery for AA may lead to complications (in 28% to 29% of cases) such as abscess formation, appendiceal perforation, systemic septic complications, wound infections, adhesions, bowel obstruction, and pulmonary complications from general anesthesia [2]. Appendectomy constitutes about 10% of all abdominal surgeries worldwide, with an associated mortality of 0.1% to 5% [1,3]. Therefore, the immediate diagnosis and surgery of AA are of grave significance.

Obstruction of the appendicular lumen, regardless of the cause, is followed by polymorphous leukocyte infiltration of the muscularis mucosae, along with edema and separation of the muscle fibers of the muscularis externa, which is the widely stated pathogenesis of AA [4]. Inflammation of the adjacent peritoneum gives rise to pain localized in the lower right abdominal region [5]. The systemic inflammatory response causes neutrophilia and lymphocytopenia, increasing the neutrophil-to-lymphocyte ratio (NLR) value, a marker of inflammation in many acute inflammatory conditions [5, 6]

The diagnosis of AA is based on clinical examination, history, and investigations such as a complete blood count (CBC), ultrasound abdomen (USG), and computed tomography (CT) scan [6,7]. A study in the UK reported the diagnostic certainty using the Alvarado scoring system to range from 70% to 80%, but the diagnosis of AA remains doubtful in about 30% to 40% of cases [8]. Conditions commonly mistaken for AA due to the non-specificity and overlap of signs and symptoms with other gastro-intestinal and gastro-urinary diseases include mesenteric lymphadenitis, constipation, gastroenteritis, obstructive hernia, orchitis, urinary tract infections, and gynecological diseases such as ovarian cysts and pelvic inflammatory disease [9]. Prospective studies indicate that the Alvarado scoring system alone is not sufficient to accurately diagnose AA [10].

A comprehensive systematic review and meta-analysis on the diagnostic precision of C-reactive protein (CRP), white blood cell count (WBC), and procalcitonin for potential AA concluded that the diagnostic efficacy of the aforementioned variables is limited [11]. Tools such as CT scans and USG are costly as well as unavailable at the periphery healthcare units, where the diagnosis of AA is made clinically in correlation to the baseline investigations [6]. Thus, the development of new biological markers for improving clinical decision-making is highly desirable. In a review of randomized trials, the authors suggested the use of the Alvarado and RIPASA scoring systems, with a need for supplementary tools to acquire confident diagnoses, especially in hospitals of developing countries and rural hospitals [12]. The objective of this study was to assess the diagnostic accuracy of the NLR as a biological marker for AA.

## Materials and methods

This cross-sectional descriptive research was carried out on patients with preliminary diagnoses of AA in the surgical wards of Ayub Teaching Hospital, Abbottabad, from July to December 2022. The institutional ethical review committee of Ayub Medical Teaching Institution, Abbottabad, issued approval RC-2022/EA-01/018. Patients were chosen via the non-probability consecutive sampling technique. After taking informed consent from 108 patients initially diagnosed with AA, only 99 of them were included. Nine patients were excluded (two pregnant ladies, three conservatively managed patients, and a specimen of four patients were not received at the histopathology laboratory of the hospital). The sample size was calculated using the sensitivity and specificity formula for sample size. Taking the prevalence of AA diagnosed via NLR at 69.95%, sensitivity and specificity of NLR > 2.4 for the diagnosis of AA at 70% and 43%, respectively, and precision of 18%, the calculated sample size was 97 patients (108 patients with a 10% dropout rate) [13]. Data was collected regarding the socio-demographic parameters of the patients, and the signs and symptoms were noted from the patient’s medical record books after the on-duty surgeons obtained a detailed medical history and underwent a thorough clinical examination. Relevant investigations were ordered as per the hospital protocols, including preoperative CBC, urine R/E, and ultrasound of the abdomen and pelvis to rule out other causes such as mesenteric adenitis and ovarian torsion, as they also mimic AA. The percentage neutrophil count and percentage lymphocyte count were recorded from the CBC report, and the NLR value was then calculated from them. Postoperatively, appendectomy specimens were sent to the Ayub Teaching Hospital/Medical College laboratory for histopathology examination (the gold standard for diagnosis of AA). A consultant clinical histopathologist examined and reported the histopathology of the specimen after processing it as per their protocols to make definitive diagnoses. The data was analyzed on the Statistical Package for Social Sciences (SPSS), version 25.0 (IBM Corp., Armonk, NY). Receiver operating characteristic (ROC) analysis was done to identify the threshold value of NLR. Validity parameters were calculated for the NLR to quantify its utility in the diagnosis of AA. A p-value <0.05 was deemed statistically meaningful.

## Results

In this study, we enrolled a cohort of 99 patients, with a mean age of 23.81 ± 10.35 years. Analysis of patient demographics revealed a predominant male representation, comprising 58.60% of the cohort. Additionally, the majority of participants hailed from urban areas, constituting 55.60% of the total study population. Table 1 provides a detailed breakdown of the socio-demographic parameters, categorizing age into distinct brackets. Notably, 82.8% of the participants were under 30 years of age, underscoring the predominantly youthful nature of the cohort. Conversely, only a nominal 4% were above 50 years of age. The gender distribution in the study cohort revealed a male-to-female ratio of 1.41:1, with males representing 58.60% and females representing 41.40% of the total sample. Furthermore, the urban-to-rural residence ratio stood at 1.25:1, with 55.60% of participants residing in urban areas and 44.40% in rural settings. These findings collectively provide a nuanced understanding of the study population's composition, highlighting age, gender, and residential distribution.

**Table 1:** Socio-demographic parameters of the participants

Patient characteristics	Categories	Frequency (percentage)
Age in years (Mean ± SD 23.81 ± 10.35)	< 10 years of age	10 (10.1%)
	11 to 20 years	29 (29.3%)
	21 to 30 years	43 (43.4%)
	31 to 40 years	13 (13.1%)
	41 to 50 years	01 (01%)
	> 51 years of age	03 (03%)
Gender (Male:Female = 1.41:1)	Male	58 (58.60%)
	Female	41 (41.40%)
Residence (Urban:Rural = 1.25:1)	Urban	55 (55.60%)
	Rural	44 (44.40%)

**Figure 1:**
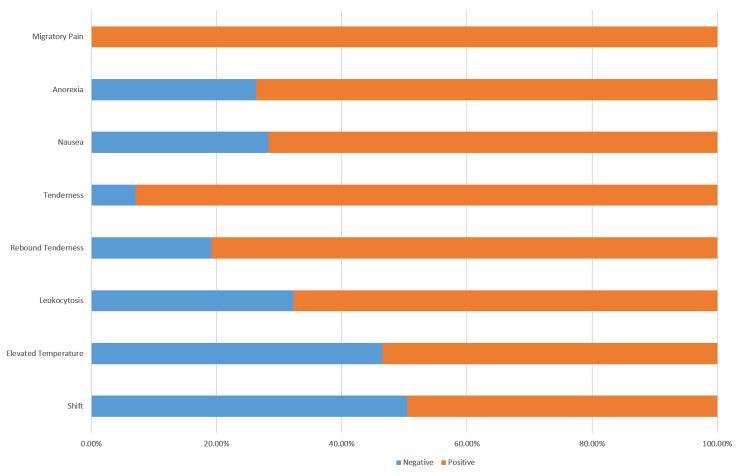
Alvarado scoring system parameters in the study participants

In our examination, we meticulously evaluated the parameters of the Alvarado scoring system, and the results are succinctly illustrated in Figure 1. These findings underscore the ubiquity of migratory abdominal pain and highlight the diverse presentation of associated signs and symptoms within our patient cohort manifested with varying frequencies. The data presented in this figure enhances our understanding of the clinical features observed in individuals undergoing assessment with the Alvarado scoring system. The Alvarado scoring system parameters, detailed in Figure 1, provide a quantitative breakdown of the frequency of occurrence of the key clinical indicators.

Preoperative CBC reports were obtained and the data regarding the percentage neutrophils count and percentage lymphocyte count were recorded and used to calculate NLR value. The NLR was calculated to have a mean ± SD of 5.80 ± 5.23 as shown in Table 2.

**Table 2:** Descriptive statistics of percentage neutrophils and lymphocyte count and NLR NLR: neutrophil-to-lymphocyte ratio

	Mean	SD	Min	Max
Percentage neutrophils count	73.10	12.72	36.30	94.00
Percentage lymphocyte count	20.62	11.32	3.10	55.40
Neutrophil-to-lymphocyte ratio	5.80	5.23	0.65	29.50

**Figure 2:**
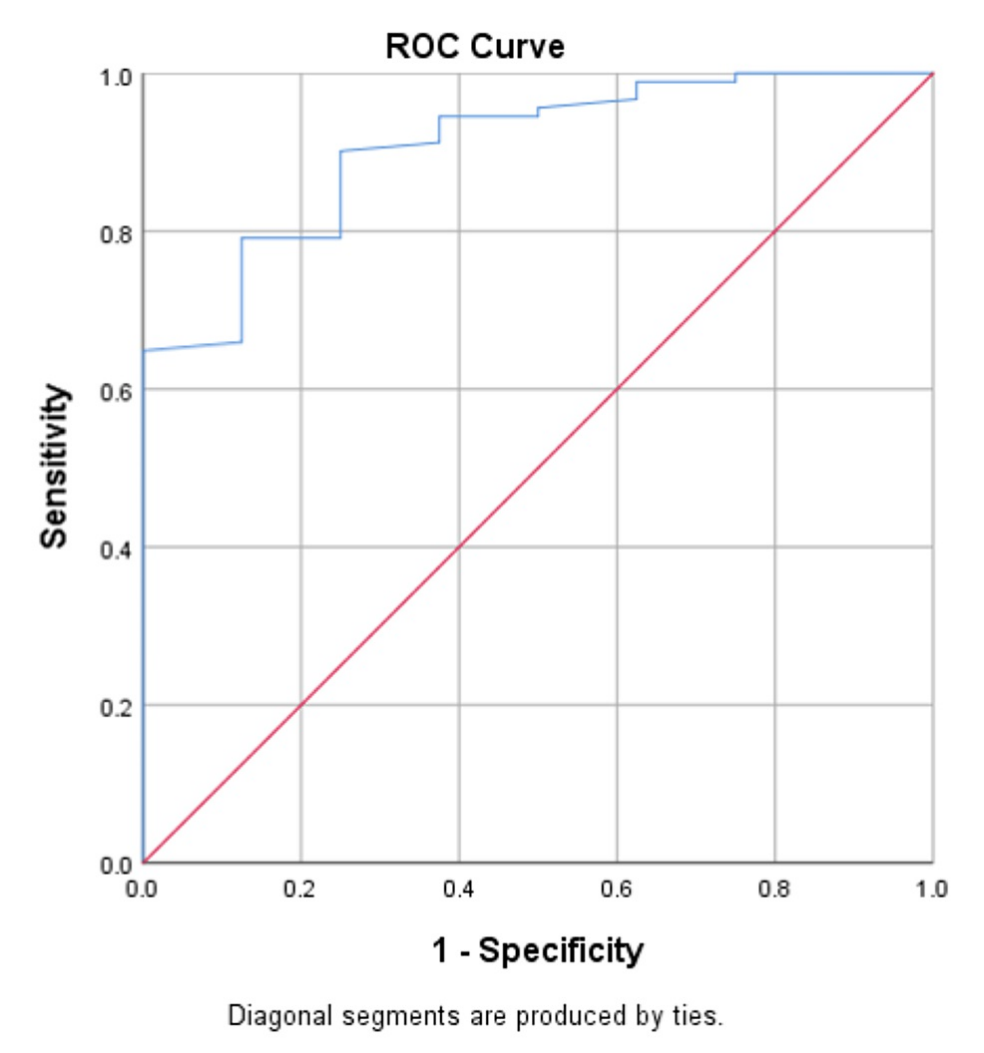
ROC curve for estimating the cut-off value for NLR ROC: receiver operating characteristic; NLR: neutrophil-to-lymphocyte ratio

In our rigorous examination, we employed ROC analysis to ascertain the optimal threshold value of NLR for the diagnosis of AA, as delineated in Figure 2. The ROC analysis determined a cut-off value of 2.49 for a positive appendectomy, revealing a sensitivity of 71.4% and 1-specificity of 12.5%. The area under the curve demonstrated a robust 90.6% (95% confidence interval {CI} 0.818-0.994, p<0.001).

Subsequently, we explored the correlation between NLR and histopathology examination of the appendix. Impressively, 91.9% of patients exhibited histopathological evidence of inflammation (positive appendectomy), while 8.1% displayed a normal appendix, contributing to a noteworthy 8.1% negative appendectomy rate, as elucidated in Table 3.

**Table 3:** Comparison of NLR with histopathology of the appendix specimen NLR: neutrophil-to-lymphocyte ratio; AA: acute appendicitis

NLR ≥ 2.5 (Indicated positive cases of AA)	Histopathology confirmed the cases			Total
		Yes	No	
	Yes	(True Positive) 65	(False Positive) 1	66
	No	(False Negative) 26	(True Negative) 7	33
Total		91	8	99

At the identified NLR cut-off value of 2.49, we computed the validity parameters, demonstrating statistical significance with a p-value of 0.001 on Pearson’s chi-square test. The sensitivity stood at 71.43%, indicating the proportion of true positives accurately identified. Furthermore, the specificity reached 87.5%, reflecting the accuracy in identifying true negatives. Our analysis revealed a positive predictive value (PPV) of 98.46%, underlining the reliability of a positive result, and a negative predictive value (NPV) of 21.21%, portraying the probability of a true negative result. Finally, the diagnostic accuracy of 72.73% emphasizes the overall precision of our diagnostic approach. These results underscore the utility of NLR as a diagnostic tool for AA, offering both sensitivity and specificity and providing valuable insights into the predictive power of this metric in clinical practice.

## Discussion

Men exhibit a higher prevalence of appendicitis compared to women, with a male-to-female ratio of 1.5:1 [14,15]. The lifetime risk for the development of AA is higher among males (8.6%) than in females (6.7%) [16]. It is during the second and third decades of life that the incidence peaks, categorizing AA as a condition primarily afflicting individuals in their early adulthood [17]. In our study group, the majority of the patients were young, as around 72% were within the second and third decades of their lives. The mean age of patients was 23.81 ± 10.35 years. This aligns closely with the mean age of patients diagnosed with AA as reported in the literature, typically falling within the range of 23-29 years [13,17,18]. Of the 91 confirmed cases of AA on histopathology in our study, 54 (59.34%) were male while 37 (40.66%) were female, making a male-to-female ratio of 1.49:1. Kucuk E investigated variations in the NLR among 241 patients diagnosed with AA [14]. The observed gender distribution indicated a ratio of 1.5 males to 1 female among individuals seeking medical attention in the emergency department with signs and symptoms suggestive of AA.

The mean negative appendectomy rate for both genders, as stated in different studies, is variable, being as low as 8.2% to 15-30% [4,6]. The observed rate of negative appendectomy in our study stood at 8.1%. This relatively low incidence can be attributed to heightened vigilance among our surgical residents during the initial phases of data collection. Recognizing the significance of minimizing unnecessary appendectomy, the residents exercised additional caution by conducting thorough investigations and seeking senior consultations before opting for surgical intervention. This exemplifies the manifestation of Hawthorne bias [19]. However, it is crucial to note that this observed trend holds limited relevance to our study objectives. Our research focus does not delve into evaluating the diagnostic quality within a tertiary care setting, and we deliberately excluded subjective parameters susceptible to clinician influence. Consequently, the observed reduction in negative appendectomy rates, while interesting, is deemed statistically insignificant within the context of our study.

The existing literature underscores various studies exploring distinct laboratory markers, including CRP, mean platelet volume (MPV), total leucocyte count (TLC), red blood cell distribution width, interleukin 6, and procalcitonin, as tools for diagnosing AA. However, among these markers, NLR emerges as particularly noteworthy, demonstrating superior diagnostic accuracy compared to other individual laboratory markers alone [1,20]. The dynamics of NLR in AA involve an increase in neutrophil percentage and a decrease in lymphocyte percentage, which together serve as a diagnostic marker [21]. It is essential to acknowledge, however, that individual variations in inflammatory responses may lead to differences in NLR among patients [14]. In our research, we established a threshold of 2.49 for the NLR through ROC analysis, yielding a sensitivity of 71.4% and a 1-specificity of 12.5%, with an area under the curve of 90.6% (95% confidence interval {CI} 0.818-0.994, p<0.001) for the diagnosis of AA. The validity parameters for NLR at a cut-off value of 2.49 were calculated from a 2x2 contingency table, taking histopathology diagnosis as the gold standard. The sensitivity, specificity, and diagnostic accuracy calculated were 71.43%, 87.5%, 98.46%, 21.21%, and 72.73%, respectively.

A study from England reported a cut-off value of NLR of 4.2 (AUC = 0.695 {CI = 0.619 - 0.771}) for diagnosing AA, with a sensitivity of 79.5% and specificity of 67.0% [22]. They suggested a limited additional benefit of using NLR instead of leukocytosis. However, a study from Turkey concluded that NLR provided better information for the diagnosis of AA as compared to leukocytosis [23]. The AUC for NLR > 8 in their study was 0.923, compared to 0.667 for leukocyte count. They reported an NLR sensitivity of 100% and a specificity of 81.6% respectively. Similar results were published by a study from Malaysia, the authors identified that an NLR with a cut-off point of ≥ 3.11 could effectively differentiate between normal appendix and AA patients [18]. The reported sensitivity was 75.23%, and the specificity was 68.70%. They concluded that NLR is a reliable and useful adjunct to the clinical examination for diagnosing AA. A meta-analysis of 17 studies found that the cut-off value of NLR for diagnosing AA was 4.7 (AUC=0.96), with a sensitivity of 88.89% and specificity of 90.91% [24]. NLR >4.7 was reported to be a predictor of AA (OR:128, P < 0.0001). Kostakis et al. contributed valuable insights into the diagnostic utility of NLR in AA [25]. They reported that an NLR ≥ 3 demonstrated a high level of diagnostic accuracy, with a sensitivity of 90%, specificity of 88%, and an overall accuracy of 89%. Moreover, when the NLR threshold was increased to ≥ 3.5, the diagnostic performance improved, showing a sensitivity of 90%, specificity of 90%, and accuracy of 90%. Pereira et al. identified a significant relationship between NLR and AA at a cut-off NLR value of >2.4 (p = 0.0001), demonstrating a sensitivity of 70.1% and specificity of 43.2% [13]. Notably, an NLR > 4.3 was specifically associated with complicated appendicitis cases, exhibiting a sensitivity of 72.25% and specificity of 54.09% [13].

These findings collectively emphasize the potential of NLR as a valuable diagnostic tool in the context of AA, with varying cut-off values providing clinicians with flexibility in utilizing this marker based on specific diagnostic requirements. The diagnosis of AA can be made confidently with an excellent history and proper examination in correlation with CBC, USG abdomen, and urine R/E [22]. The NLR value can be helpful in the diagnosis of doubtful cases of right iliac fossa (RIF) pain as well as at the periphery healthcare units, where the diagnosis of AA is made clinically in correlation to the baseline investigations due to the unavailability of sophisticated tools [11,12,24].​​* *This simple tool can be used as it is easily available, cheap, and easy to perform and interpret. For an exploration of the optimum NLR value and a thorough evaluation of its accuracy, it is imperative to have randomized studies conducted under controlled circumstances. The primary limitation of our study was that we lacked the data of patients with suspicious abdominal findings who did not undergo surgery.

## Conclusions

Elevated NLR may help in the definitive diagnosis of AA, however, a normal value for NLR does not rule out the diagnosis. We report a strong correlation between NLR and the diagnosis of AA and therefore suggest that NLR should be utilized as a complementary biomarker to clinical examination, aiding in the diagnosis of AA.
